# Spin-orbit Rabi oscillations in optically synthesized magnetic fields

**DOI:** 10.1038/s41377-023-01238-8

**Published:** 2023-08-28

**Authors:** Guohua Liu, Xiliang Zhang, Xin Zhang, Yanwen Hu, Zhen Li, Zhenqiang Chen, Shenhe Fu

**Affiliations:** 1 Department of Optoelectronic Engineering, Jinan University, Guangzhou, 510632 China; 2 Guangdong Provincial Key Laboratory of Optical Fiber Sensing and Communications, Guangzhou, 510632 China; 3 Guangdong Provincial Engineering Research Center of Crystal and Laser Technology, Guangzhou, 510632 China

**Keywords:** Optical physics, Optical physics

## Abstract

Rabi oscillation has been proven to be one of the cornerstones of quantum mechanics, triggering substantial investigations in different disciplines and various important applications both in the classical and quantum regimes. So far, two independent classes of wave states in the Rabi oscillations have been revealed as spin waves and orbital waves, while a Rabi wave state simultaneously merging the spin and orbital angular momentum has remained elusive. Here we report on the experimental and theoretical observation and control of spin–orbit-coupled Rabi oscillations in the higher-order regime of light. We constitute a pseudo spin-1/2 formalism and optically synthesize a magnetization vector through light-crystal interaction. We observe simultaneous oscillations of these ingredients in weak and strong coupling regimes, which are effectively controlled by a beam-dependent synthetic magnetic field. We introduce an electrically tunable platform, allowing fine control of transition between different oscillatory modes, resulting in an emission of orbital-angular-momentum beams with tunable topological structures. Our results constitute a general framework to explore spin–orbit couplings in the higher-order regime, offering routes to manipulating the spin and orbital angular momentum in three and four dimensions. The close analogy with the Pauli equation in quantum mechanics, nonlinear optics, etc., implies that the demonstrated concept can be readily generalized to different disciplines.

## Abstract (web summary)

The spinor evolves along a direction perpendicular to synthetic magnetic field **B** and state vector **S**, resulting in Rabi oscillatory modes. Trajectories in phase matching and mismatching conditions are presented for positive and negative crystals.

## Graphical abstract

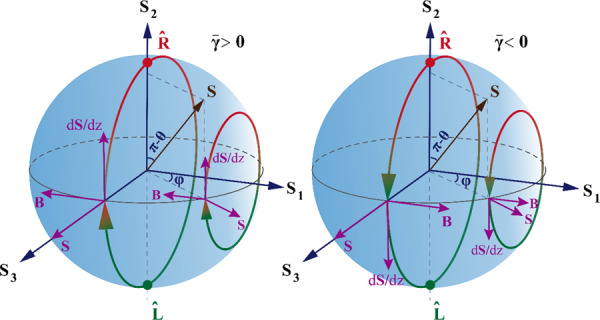

## Introduction

Rabi oscillation expresses a phenomenon that a quantum wave packet initially nested in a ground state of a two-level system can be excited by an external magnetic field to another state and then returns to its origin after a circle evolution^1^. It has been proven to be one of the cornerstones of quantum mechanics, triggering various applications ranging from nuclear magnetic resonance imaging and spectroscopy to quantum information processing^2^. Even though it has been known for a long time since its discovery in 1937, its study remains to uncover new physics^3–5^. Substantial investigations on the Rabi oscillations have been carried out over the years in different settings, including atomic and molecular physics^6,7^, acoustics^8^, and optics^9–11^, by taking advantage of the analogous spin–orbit couplings^12–14^. In optics, spin–orbit coupling refers to an interaction between the spin and orbital angular momentum (SAM and OAM)^14^. While the SAM is associated with circular polarization characterized by a spin number *ϱ*^15^, the OAM is generally related to a helical wavefront featured by a topological charge ${\ell}$^16^. The SAM–OAM interplays have induced striking phenomena, including the optical Hall effects^17–25^ and mutual conversions between SAM and OAM^26–29^. In this article, we exploit the spin–orbit coupling, unraveling the Rabi oscillating state of light that simultaneously merges the spin and orbital angular momentum. This new form of Rabi oscillations with both SAM and OAM remains unknown to our knowledge. We study this effect through an optically synthesized magnetic field, acting on a mutual beam comprising the superposition of both the spin and orbital angular momentum.

In this context, the spin–orbit coupling for the Rabi oscillations is closely related to the synthesized magnetic field. This is even highlighted by other dynamical spin–orbit phenomena in systems driven by the synthesized magnetic fields^30–35^. The concept of what constitutes the magnetic field has expanded over the years. However, such an expansion maintains the fact that the spin–orbit coupling process is described by the pseudo spin-1/2 model. Among others, we remind the spin dynamics of exciton-polaritons in microcavity structures, where transverse splitting of polaritons constitutes the effective magnetic field^20,30,31^. The three-wave-mixing process in nonlinear optics emulates the spin-1/2 system, where nonlinear coupling and inherent phase mismatch constitute the magnetic field equivalent^32–35^. It is indicated that the synthesized magnetic fields are merely associated with structured materials^30–37^. The material dependence of the magnetic field provides limited degrees to control the spin–orbit coupling.

We present a distinct pseudo spin-1/2 formalism in the higher-order optical regime and constitute the magnetic field through light-crystal interaction. The synthesized magnetic field is fully controlled by either structuring the light beam or engineering the crystal. Using a beam-dependent synthetic magnetic field, we demonstrate the concept of spin–orbit Rabi oscillations both in the strong and weak coupling regimes. The beam comprises the superposition of two spin–orbit eigenstates, which are defined as spin-up and spin-down equivalents of the two-level system^38^. We demonstrate a tunable platform, enabling electrically engineering of the spin–orbit Rabi oscillations in the phase mismatching regime. This electrical knob permits to finely tune the magnetic field and hence controls transitions between different modes, allowing a vertical emission of OAM beam with tunable topological structures. The formalism constitutes a general framework for spin–orbit dynamics with the synthesized magnetic fields. Since the setting is equivalent to those described by the Pauli equations, such as in quantum mechanics and nonlinear optics, our results open new possibilities for spinor manipulation in the higher-order regime. Since structured light carrying the SAM and OAM has attracted attention in a broad range^39–43^, our results can find potential applications in classical and quantum optics^44,45^.

## Results

### Observations of spin–orbit Rabi oscillations

**Fig. 1:**
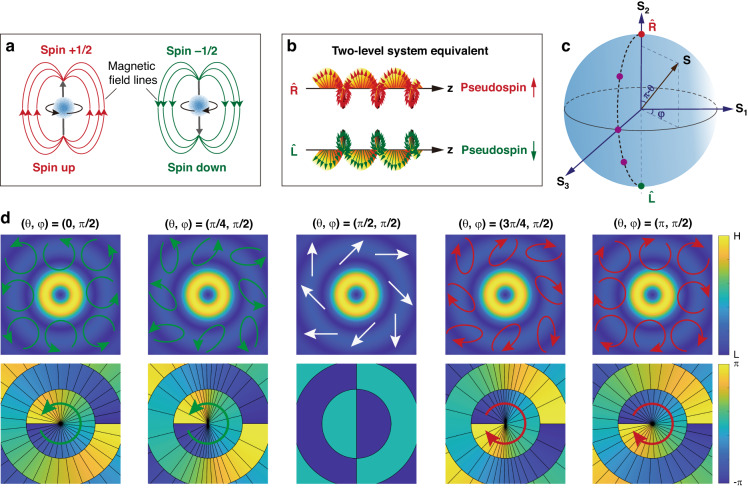
The equivalent two-level system in the higher-order regime. **a** The original spin-1/2 setting describes the spinnings of a quantum particle in a driven magnetic field, where the spin up $(\varrho =+1/2)$ and spin down $(\varrho =-1/2)$ constitute the two-level eigenstates. **b** In the higher-order optical regime, the eigenstates $\hat{R}$ and $\hat{L}$ simultaneously coupling with the SAM and OAM are defined as the spin up $(-\varrho {\mathscr{+}}{\mathscr{l}}{\mathscr{)}}$ and spin down $(+\varrho {\mathscr{-}}{\mathscr{l}}{\mathscr{)}}$ equivalents in a pseudo spin-1/2 system, respectively. These pseudo spin up and spin down are coupled by a synthetic magnetic field. **c** The higher-order Poincaré sphere is introduced to represent the spin–orbit states, with two poles denoting the eigenstates. **d** Typical states mapped on the sphere (marked points in **c**) showing spatial variations of polarization (upper panels) and phase (bottom panels) distributions with polar angle. The arrows in the upper panels denote polarizations; while the black lines in the bottom panels represent the phase contours

We start by performing experiments to observe these phenomena. We hence constitute the pseudo spin-1/2 model in the higher-order regime by defining a mutual beam comprising the superposition of two eigenstates^23^

1 $$\begin{array}{c}\hat{R}={{\exp }}\left(+i{\mathscr{l}}\phi \right)\left(\hat{x}-i\hat{y}\right)/\sqrt{2}\\ \hat{L}={{\exp }}\left(-i{\mathscr{l}}\phi \right)\left(\hat{x}+i\hat{y}\right)/\sqrt{2}\end{array}$$

where $\hat{x}$ and $\hat{y}$ are unit vectors with respect to coordinates *x* and *y*, respectively, and $\phi ={{\arctan }}(y/x)$. The eigenstates couple with both the intrinsic spin and orbital angular momentum. A superposition of $\hat{R}$ and $\hat{L}$ leads to a mutual beam which is, therefore, spin–orbit coupled and is featured by a spatially varying polarization with distribution determined by weights Φ_*R*_ and Φ_*L*_, respectively^39,46^. We unravel the similarity between the spin–orbit state of light and the spinning of a quantum particle in the spin-1/2 model^38^ by mapping dynamic states onto the higher-order Poincaré sphere^39^ and Bloch sphere (both spheres share SU(2) structure), respectively. The north and south poles of the Bloch sphere represent two eigenstates, corresponding to opposite spinnings of a quantum particle known as the purely spin up $({\varrho} =+1/2)$ and spin down $(\varrho =-1/2)$ in system subjected to a magnetic field (Fig. 1a). In the higher-order framework, we similarly define $\hat{R}$ and $\hat{L}$ as the pseudo spin up $\left(-\varrho \,+\,{\mathscr{l}}\right)$ and spin down $(+\varrho {\mathscr{-}}{\mathscr{l}}{\mathscr{)}}$ states located at the poles of the Poincaré sphere (Fig. 1b). Note that the constituted pseudo spin system is distinct from conventional spin settings by a higher-order topological charge and is driven by a synthesized magnetic field. The spinor state $({\Phi }_{R};{\Phi }_{L})$ of the mutual beam can be mapped onto the higher-order sphere (Fig. 1c), with parameters *θ* and *φ* denoting polar and azimuthal angles of the sphere, respectively. In this case, Φ_*R*_ and Φ_*L*_ are written in a normalized form as ${\Phi }_{R}={{\sin }}\left(\theta /2\right){{\exp }}(+i\varphi /2)$ and ${\Phi }_{L}={{\cos }}\left(\theta /2\right){{\exp }}(-i\varphi /2)$. Figure 1d depicts typical spinors at a longitude of the first-order sphere, showing simultaneous variations of the spin (polarization) and orbital (phase) states with *θ*.

**Fig. 2:**
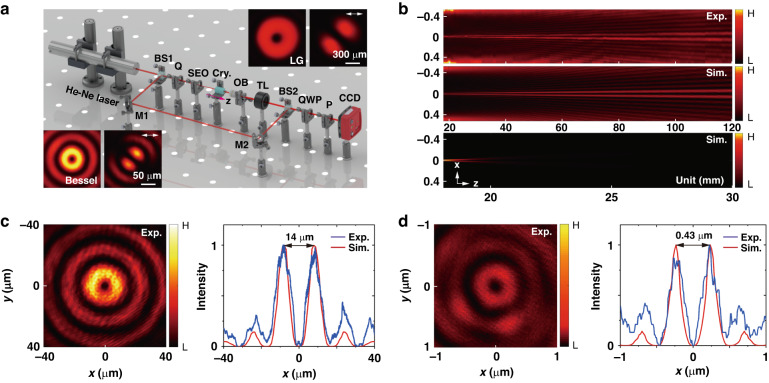
Experimental configuration for measuring the spin–orbit Rabi oscillations. **a** Experimental setup. BS beam splitter, Q q-plate, SEO sharp-edge obstacle (see Materials and methods for more details); M mirror, OB objective lens, TL tube lens, QWP quarter wave plate, P polarizer, CCD charge-coupled device. The upper inserts display the LG beam that carries a typical spin–orbit state located at $\left(\theta ,\varphi \right)=(\pi /2,\pi /2)$. The inserts at the bottom depict the generated Bessel beam while maintaining the same state as the LG beam. **b** The experimental (upper panel) and numerical (middle panel) intensity distributions of the first-order (${\mathscr{l}}{\mathscr{=}}1$) Bessel beam in the *x*–*z* plane. The beam is generated by the SEO of radius *ρ* = 400 μm. In comparison, the bottom panel shows the numerical intensity distribution of the diffracting LG beam with the same initial beam width. **c**, **d** Experimental and simulated intensity distributions of the generated Bessel beams at the input ends of the crystals for two parameters: **c** *r*_0_ = 3.5 μm; **d** *r*_0_ = 110 nm. In the color bars, L: low; and H: high

We implement experiments to reveal the beam-dependent spin–orbit Rabi oscillations. This requires generating propagation-invariant spin–orbit light with tunable beam size. Figure 2a depicts an experimental setup for measuring the spin–orbit Rabi oscillations. Specifically, a space-variant q-plate with a topological number of *q* = 1/2 is used to transform an incident linearly polarized He–Ne laser beam (*λ* = 632.8 nm) into a diffracting spin–orbit Laguerre–Gaussian (LG) beam, see upper inserts in Fig. 2a. Details for initial spinor preparation and manipulation refer to materials and methods. The generated LG beam then passes through a self-created sharp-edge element (SEO), resulting in the first-order (${\mathscr{l}}=1$) Bessel beam denoted as ${J}_{{\mathscr{l}}}(r/{r}_{0})$, where ${J}_{{\mathscr{l}}}$ is the Bessel function of order ${\ell}$, $r=\sqrt{{x}^{2}+{y}^{2}}$ and *r*_0_ features the Bessel beam size. It maintains the same spin–orbit state (see insets at the bottom in Fig. 2a), and its size can be flexibly controlled by the radius *ρ* of the element. More details about the SEO element refer to the materials and methods, as well as Supplementary Section A. In order to characterize the Bessel beam, a microscopic system comprising an objective lens, a tube lens, and a charge-coupled device (CCD) with a pixel size of 1.4 μm is utilized to properly magnify the beam. The objective lens is mounted onto a precise electric-control stage that can be moved along the distance. The nondiffracting property of the generated Bessel beam by a typical SEO of radius *ρ* = 400 μm is observed both experimentally and numerically, see Fig. 2b. Note that in practice, a Gaussian window is utilized to truncate the ideal Bessel beam, resulting in a Bessel-Gaussian beam which exhibits weakly diffracting property during propagation (Fig. 2b). However, considering serious diffraction of the LG beam with identical beam width (see the bottom panel in Fig. 2b), as well as relatively small coupling length of the crystal, the diffracting of the Bessel–Gaussian beam is negligible. To observe the Rabi oscillations, a number of crystals used to couple the two eigenstates are appropriately prepared and aligned such that the light travels along their optical axis, denoted as the *z*-axis. Detail about the experimental alignment of the crystal refers to materials and methods. We define anisotropic degree of the crystal as $\bar{\gamma }=({\gamma }_{x}+{\gamma }_{y})/2$, where ${\gamma }_{j}=1-{\epsilon }_{j}/{\epsilon }_{z}$ (here *j* = *x* or *y*) with $\hat{\epsilon }$ being a dielectric tensor of the crystal. The parameter $\bar{\gamma }$ is either positive or negative, allowing control of the spin–orbit coupling by engineering polarity in the crystal. Owing to spin–orbit coupling, the mutual beam emerging from the crystal is expected to accumulate a *z*-dependent helical wavefront carrying nonzero OAM. We hence use a reference plane-wave beam to interfere with it, measuring the orbital angular momentum oscillations.

**Fig. 3:**
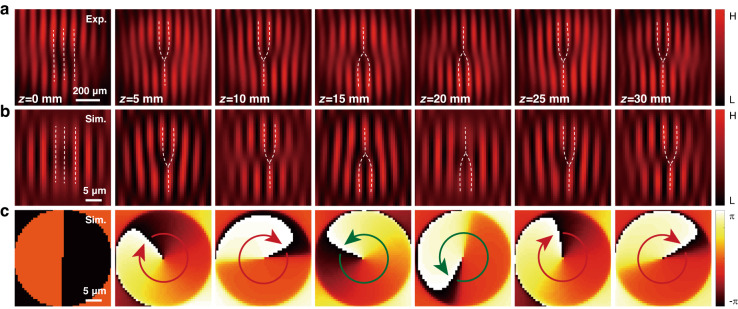
Observation of the spinor oscillation in the weak coupling regime. The spinor starts from an equatorial position $(\theta =\pi /2,\varphi =\pi /2)$ where the spin and orbital angular momentum are zeros. A typical parameter of the nondiffracting mutual beam is chosen as *r*_0_ = 3.5 μm. In the presence of synthetic magnetization ($\bar{\gamma }=0.19$), the spinor evolves adiabatically from the equator toward the north pole, giving rise to a separation between the spin and orbital angular momentum. The Rabi oscillation is manifested partially by the separated orbital-angular-momentum oscillation along with the coupling length. **a**, **b** The interferograms recorded at different coupling lengths: **a** experiments; **b** simulations. **c** Simulated phase distributions of the light field at the corresponding coupling lengths. Panels in **a** (**b**, **c**) share the same scale, with a scale bar of 200 μm (5 μm)

The first experiment is performed with a c-cut yttrium vanadate bulk crystal (positive polarity $\bar{\gamma }=0.19$). We choose a typical beam parameter as ${r}_{0}\simeq 3.5\,\mu {\rm{m}}$, corresponding to a ring size of about 14 μm. The initial beam intensity is measured by the objective lens (Nikon, Plan Fluor 40×, NA = 0.75), as shown in Fig. 2c. The measured interferograms at different coupling lengths are presented in Fig. 3a. At the beginning (*z* = 0), the spinor stays at the equatorial position $(\theta =\pi /2,\varphi =\pi /2)$ of the sphere with zero orbital angular momentum, see a measurement showing regular interference fringes. After a coupling length of *z* = 5 mm, it accumulates a helical wavefront with a topological charge of ${\mathscr{l}}=1$, as indicated by a dislocation in the fringes. Afterward, a detection at *z* = 10 mm monitors the state that maintains the same topological wavefront. The measurements at *z* = 5 and 10 mm signify a forward process for a cycle evolution, while the measurements at *z* = 15 and 20 mm suggest a reverse process since the orientation of the dislocation is opposite to that forward process. The measurements at *z* = 25 and 30 mm suggest a repeated process after a cycle evolution. These observations manifest the emergence of a spinor oscillation along with the coupling length with a coupling period estimated as Λ ≈ 20 mm.

In addition to the OAM oscillation, the spin–orbit coupling process also suggests a simultaneous SAM oscillation at the mercy of total angular momentum conservation law. We observe this phenomenon by inserting a circular polarization analyzer in front of the CCD. The analyzer consists of a quarter wave plate and a horizontal linear polarizer, see the setup in Fig. 2a. With this configuration, we obtain the SAM oscillation as illustrated in Supplementary Section B, where we show the oscillations of the left- and right-handed circularly polarized components of light with the coupling length. These results, together with those in Fig. 3a, confirm a spin–orbit Rabi oscillation.

**Fig. 4:**
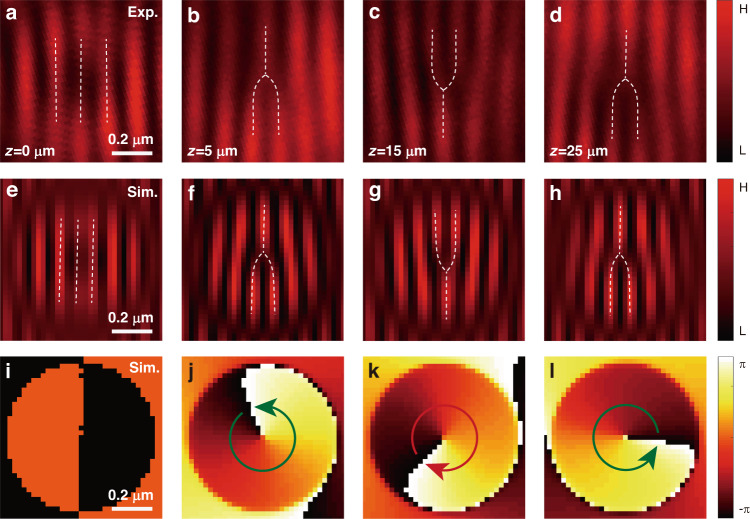
Observation of the spinor oscillation in the strong coupling regime. The giant synthetic magnetic field is achieved by a deep subwavelength beam featured by a beam parameter of *r*_0_ ≈ 110 nm. The spinor starts from the same position $(\theta =\pi /2,\varphi =\pi /2)$ and evolves rapidly along an opposite path due to the reversed crystal polarity ($\bar{\gamma }=-0.16$). **a**–**d** The resulting interferograms are recorded at different coupling lengths, whereas **e**–**h** shows the corresponding simulations. **i**–**l** The simulated phase distributions of light at the corresponding positions. All panels share the same scale. Scale bar: 0.2 μm

A subsequent experiment with a significant decrease in the beam width reveals the Rabi oscillation with a large Rabi frequency. We consider using a deep-subwavelength nondiffracting light beam. So far, it remains a challenge to generate the well-defined nondiffracting spin–orbit state on such an extremely small scale since its topological structure is usually unsustained in tightly focusing. Our self-created element is able to overcome this challenge (see Materials and Methods). We slightly modify the setup by using a high-numerical-aperture objective lens (Nikon, CFI EPI 150×, NA = 0.9) to characterize the Bessel beam at the subwavelength scale. The ring size of the doughnut beam is measured as 430 nm, approximately corresponding to a beam parameter of *r*_0_ = 110 nm; see the measured intensity distribution in Fig. 2d. Figure 4 presents the results obtained with a c-cut barium metaborate film (negative polarity $\bar{\gamma }={-}0.16$). In this scenario, the state starts from the same equatorial position, evolving along an opposite path. The initial spinor evolves into a nearly pure spin-down state (Φ_*R*_; Φ_*L*_) ≃ (0; 1) for an extremely short coupling length of *z* = 5 μm; see the result in Fig. 4b. It means that the right-handed component is converted to its counterpart. Slightly increasing the coupling length to *z* = 15 μm, we observe the spinor located at the spin-up state (Φ_*R*_; Φ_*L*_) ≃ (1; 0), causing the subwavelength beam to have a reversed topological wavefront. Such a reciprocal topology is indicated by an opposite dislocation in the interference fringes (Fig. 4c). Again, the spinor becomes spin down (Φ_*R*_; Φ_*L*_) ≃ (0; 1) when the coupling length gradually increased to *z* = 25 μm (Fig. 4d). The experimental data indicates a rather small coupling period, confirming a harmonic oscillation of the spinor with a considerably large Rabi frequency.

### A theoretical model of beam-dependent synthesized magnetic field

We present a pseudo spin-1/2 formalism to explain the beam-dependent spin–orbit Rabi oscillations. We synthesize a magnetic field equivalent that drives the spin-orbit coupling process. Specifically, we investigate the vector-wave equation $\nabla \times [{\mu }^{-1}\cdot (\nabla \times \widetilde{{\bf{E}}})]=-\hat{\epsilon }\cdot {\partial }^{2}\widetilde{{\bf{E}}}/\partial {t}^{2}$, where $\widetilde{{\bf{E}}}$ represents a light field, *μ* denotes the permeability of the crystal, and *t* is elapsed time. Considering a wave form: $\widetilde{{\bf{E}}}={\bf{E}}{{\exp }}(-i\omega t)$, where ${E}_{j}\left(x,y,z\right)={A}_{j}\left(x,y,z\right){{\exp }}(i{\beta }_{j}z)$ ($j=x,y,z$) is a component of **E**, we separate the time and space variables in the wave equation (see materials and methods). Here *ω* is a carrier frequency of light and ${\beta }_{j}=\omega \sqrt{\mu {\epsilon }_{j}}$ denotes a propagation constant of wave component in the crystal. We assume the complex envelope varies slowly with propagation distance *z* and consider transforming *A*_*x*_ and *A*_*y*_ to a rotating frame. Hence, we define ${A}_{x}={{\exp }}(i\triangle \beta \cdot z/2){\widetilde{A}}_{x}$ and ${A}_{y}={{\exp }}(-i\triangle \beta \cdot z/2){\widetilde{A}}_{y}$, respectively, where $\triangle \beta ={\beta }_{y}-{\beta }_{x}$ is a phase mismatch parameter arising from the beating between *A*_*x*_ and *A*_*y*_ during propagation. In the rotating frame, a general equation governing the spin-orbit coupling is obtained as

2 $$i\frac{\partial }{\partial z}\left[\begin{array}{c}{\widetilde{A}}_{x}\left(z\right)\\ {\widetilde{A}}_{y}\left(z\right)\end{array}\right]=\left({{\bf{H}}}_{0}+{\bf{H}}\right)\left[\begin{array}{c}{\widetilde{A}}_{x}\left(z\right)\\ {\widetilde{A}}_{y}\left(z\right)\end{array}\right]$$

where ${{\bf{H}}}_{0}={(2\bar{\beta })}^{-1}[-{\nabla }_{\perp }^{2}+\bar{\gamma }{\nabla }_{{xx}},0;0,-{\nabla }_{\perp }^{2}+\bar{\gamma }{\nabla }_{{yy}}]$ and ${\bf{H}}=[{H}_{11},{H}_{12};{H}_{21},{H}_{22}]$ represent equivalents of the intrinsic and interactive Hamiltonians, respectively, with ${H}_{11}=\triangle \beta /2$, ${H}_{12}=\bar{\gamma }{\nabla }_{{yx}}/(2\bar{\beta })$, ${H}_{21}=\bar{\gamma }{\nabla }_{{xy}}/(2\bar{\beta })$, and ${H}_{22}=-\triangle \beta /2$, respectively. Here we define $\bar{\beta }=({\beta }_{x}+{\beta }_{y})/2$, and ${\nabla }_{\perp }^{2}$ represents a transverse momentum operator. We find that the interactive Hamiltonian coupling the two components is closely related to spatial gradient operators: ${\nabla }_{{xy}}={\partial }^{2}/(\partial x\partial y)$ and ${\nabla }_{{yx}}={\partial }^{2}/(\partial y\partial x)$ (${\nabla }_{{xy}}={\nabla }_{{yx}}$). That means the spin-orbit coupling can be spatially engineered by shaping the beam. This finding of beam-dependent spin–orbit coupling is fundamentally different from those being material-dependent^30–37^. Even though optical spin–orbit couplings have been extensively investigated in anisotropic crystals, see^47–51^ among others, this beam-dependent phenomenon is not reported. We express the mutual beam as $\widetilde{{\bf{A}}}\left(x,y,z\right)={\widetilde{A}}_{0}(x,y,z)[{\Phi }_{R}\left(z\right)\hat{R}+{\Phi }_{L}\left(z\right)\hat{L}]$, where ${\Phi }_{R}\left(z\right)$ and ${\Phi }_{L}\left(z\right)$ denote the *z*-dependent coefficients of weights on $\hat{R}$ and $\hat{L}$, respectively.

Equation (2) is transformed to a spin-1/2 form using the pseudo spin description. Hence we rewrite the Hamiltonian equivalent according to a transformation from the Cartesian basis to the circular basis. Note that while the index of *x*, *y*, *z* denotes spatial coordinates in the Cartesian system, the subscript index of 1–3 is adopted to represent three components in the circular basis. The resulting intrinsic Hamiltonian is then given by: ${{\bf{H}}}_{0}^{{\prime} }={\rm{T}}{{\bf{H}}}_{0}{{\rm{T}}}^{-1}=(\bar{\gamma }-2)/(4\bar{\beta })[{\nabla }_{\perp }^{2},0;0,{\nabla }_{\perp }^{2}]$, where ${\rm{T}}=[1,-{i;}1,i]$ is a transformation matrix; whereas the resulting interactive Hamiltonian (${{\bf{H}}}^{{\prime} }={\rm{T}}{\bf{H}}{{\rm{T}}}^{-1}$) takes the following forms: ${H}_{11}^{{\prime} }=0,$ ${H}_{12}^{{\prime} }=\Delta \beta /2-i\bar{\gamma }{\nabla }_{{yx}}/(2\bar{\beta })$, ${H}_{21}^{{\prime} }=\Delta \beta /2+i\bar{\gamma }{\nabla }_{{yx}}/(2\bar{\beta })$ and ${H}_{22}^{{\prime} }=0$. A spin-1/2 model is therefore obtained as

3 $$i\frac{\partial }{\partial z}\left[\begin{array}{c}{\Phi }_{R}\left(z\right)\\ {\Phi }_{L}\left(z\right)\end{array}\right]=\left(\frac{1}{2{\bf{M}}}{\nabla }_{\perp }^{2}{\widetilde{A}}_{0}-\frac{1}{2}\sigma \cdot {\bf{B}}\right)\left[\begin{array}{c}{\Phi }_{R}\left(z\right)\\ {\Phi }_{L}\left(z\right)\end{array}\right]$$

where ${\bf{M}}=2\bar{\beta }{\widetilde{A}}_{0}/(\bar{\gamma }-2)\cdot {\bf{I}}$ is the equivalent mass of the spinor, with **I** being the 2 × 2 identity operator and **B** is the synthetic magnetic field. *σ* is the Pauli matrix vector $\sigma =({\sigma }_{1},{\sigma }_{2},{\sigma }_{3})$. In the circular basis, it is expressed as ${\sigma }_{1}=[0,-{i;i},0]$, ${\sigma }_{2}=[\mathrm{1,0};0,-1]$, and ${\sigma }_{3}=[\mathrm{0,1};\mathrm{1,0}]$, respectively. Equation (3) exhibits an identical form to the Pauli equation: $i\dot{\Psi }=({\nabla }_{\perp }^{2}/2m-\sigma {\boldsymbol{\cdot }}{\bf{B}})\Psi$^52^, where $\dot{\Psi }$ defines the wavefunction of a spin particle of mass *m* being evolution as the time axis. The resulting magnetization vector ${\bf{B}}=({B}_{1},{B}_{2},{B}_{3})$ is given by ${B}_{1}=-\Omega$, ${B}_{2}=0$, ${B}_{3}=-\Delta \beta$, where

4 $$\Omega =\frac{\bar{\gamma }}{\bar{\beta }}\frac{\iint \left|{\nabla }_{{xy}}{\widetilde{A}}_{0}\left(x,y\right)\right|{dxdy}}{\iint \left|{\widetilde{A}}_{0}\left(x,y\right)\right|{dxdy}}$$

The averaged strength of the synthesized magnetic field is therefore obtained as $\left|{\bf{B}}\right|={({\Omega }^{2}+{\Delta \beta }^{2})}^{1/2}$. Clearly, while the two eigenstates $\hat{R}$ and $\hat{L}$ define the equivalents of the spin-up and spin-down states in the two-level system, the spatial gradient of the light field, together with the inherent phase mismatch Δ*β* and linear coupling $\bar{\gamma }$, constitutes an optical equivalent of the magnetic field. Note that the wave nature of diffraction not only breaks the spin–orbit state during propagation but also substantially weakens the spin–orbit coupling strength; see Supplementary Section C for more details about the influence of diffraction on the spin–orbit coupling. To address this issue, we exploit a *nondiffracting mutual beam* interacting with the crystal. In this scenario, Eq. (3) emulates a perfect process for the spin-orbit-coupled Rabi oscillations. The finding is essential in that, while the spin-orbit coupling is usually nonadjustable in the light-matter interaction, the synthesized magnetic field presented here can be arbitrarily controlled either by engineering the phase mismatch Δ*β* in the crystal or by structuring the light field ${\widetilde{A}}_{0}(x,y)$ in space. These different degrees of freedom allow full control of the spin–orbit couplings, leading to intriguing phenomena analogous to those reported with the Pauli equation^32,33,52,53^. The presented formalism constitutes a general theoretical framework for exploring the spin-orbit couplings in the higher-order regime.

**Fig. 5:**
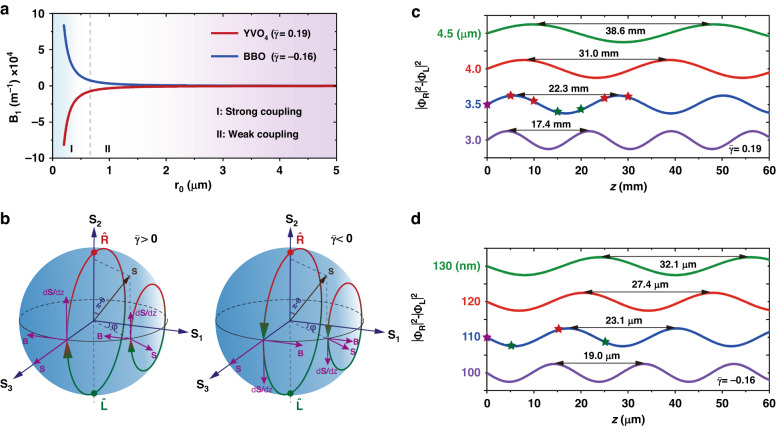
Spin–orbit Rabi oscillations induced by beam-dependent synthetic magnetic fields. **a** Relationship between the strength of the effective magnetization vector and the Bessel beam width, revealing two prominent regimes: strong and weak couplings separated at ${r}_{0}=\lambda$. **b** Geometrical representation for spinor precession about the magnetization vector **B**. The initial spinor evolves adiabatically along a direction perpendicular to **B** and **S**, resulting in Rabi oscillatory modes. Two typical evolution trajectories are presented by setting $\varphi =\pi /4$ and $\varphi =\pi /2$, both for $\bar{\gamma } > 0$ and $\bar{\gamma } < 0$. **c**, **d** Spinor oscillations with coupling length in terms of the normalized value ${\left|{\Phi }_{R}\right|}^{2}-{\left|{\Phi }_{L}\right|}^{2}$ in different cases of structured beam widths: **c** weak coupling; **d** strong coupling. The star symbols in **c** and **d** indicate experimental coupling lengths used to measure the Rabi oscillations

In the following, we use the controllable synthetic magnetic field to address the observed spin–orbit Rabi oscillations, by spatially structuring the light beam, for the fixed crystals. Particularly, under phase matching conditions (Δ*β* = 0), the synthetic magnetization vector **B** is mainly determined by the spatial gradient of the beam structure. We calculate the strength of **B** through a Bessel envelope structure ${\widetilde{A}}_{0}\left(r\right)={J}_{{\mathscr{l}}}(r/{r}_{0})$. Figure 5a presents the relationship between *B*_1_ and *r*_0_ for the positive ($\bar{\gamma }=0.19$) and negative ($\bar{\gamma }=-0.16$) crystals. Depending on the value of |**B**|, we classify the spin–orbit coupling into two prominent regimes: the strong and weak couplings separated by a critical line at ${r}_{0}\simeq \lambda$.

This result indicates that the strong coupling necessitates a subwavelength light beam which exhibits a prominent spatial gradient. The strength of **B** increases dramatically with a slight decrease of *r*_0_. When increasing *r*_0_ exceeding the wavelength, |**B**| becomes much smaller than those in the subwavelength cases and approximately invariant with *r*_0_. Figure 5a suggests a new mechanism, namely considering structured light rather than structured materials, to effectively control the strength of spin–orbit coupling.

The Rabi oscillation is a consequence of the magnetization vector acting on the spinor. The spinor evolution is realized as a precession of the state vector around the magnetic field, which is written as $d{\bf{S}}/{dz}={\bf{B}}\times {\bf{S}}$, where ${\bf{S}}=({S}_{1},{S}_{2},{S}_{3})$ denotes the spin-orbit state vector and is defined as ${S}_{\nu }={\Phi }^{\dagger }{\sigma }_{\nu }\Phi$ (with *ν* = 1, 2, 3 and $\Phi =({\Phi }_{R}{\rm{;}}{\Phi }_{L})$). The state vector evolves adiabatically in a direction perpendicular to the vectors **B** and **S**. Since the magnetization orientation is purely antiparallel ($\bar{\gamma } \,>\, 0$) or parallel ($\bar{\gamma } \,< \,0$) to *S*_1_ axis, we consider an initial state with equal weight on $\hat{R}$ and $\hat{L}$, while the state vector being parallel to *S*_3_ axis $(\theta =\pi /2,\phi =\pi /2)$. Under the action of the magnetic field, it evolves along a longitude line and toward the north pole of the higher-order sphere if the crystal polarity is positive ($\bar{\gamma }\, > \,0$), see the left panel in Fig. 5b. In the case of $\bar{\gamma }\, <\, 0$, the state vector evolves in the opposite direction along the south pole (right panel). The geometrical representations indicate resonant (phase-match) spin–orbit Rabi oscillations. Figure 5c, d depicts spinor harmonic oscillations along with *z*, represented by ${S}_{2}\left(z\right)={\left|{\Phi }_{R}(z)\right|}^{2}-{\left|{\Phi }_{L}(z)\right|}^{2}$, in the weak and strong coupling regimes, respectively. The value of *S*_2_ is between −1 and 1, which is not shown in the panels. We observe that whereas in the weak coupling regime, the relatively small synthetic magnetism leads to an oscillating period of the spinor in the order of centimetre, the giant magnetism in the strong coupling regime significantly reduces the coupling period that is three orders of magnitude smaller than the former one. We take the experimental beam parameters *r*_0_ = 3.5 μm and *r*_0_ = 110 nm as examples of the weak and strong coupling regimes. The corresponding oscillation periods are calculated as Λ = 22.3 mm and Λ = 23.1 μm, which are approximately in accordance with the experimental results, see experimental data of coupling lengths indicated by stars in Fig. 5c, d, respectively. To further test the experimental results, we perform simulations based on Eq. (2); see Fig. 3b, c for weak coupling and Fig. 4e–l for strong coupling. In both cases, the simulated fringes and phase mappings at the corresponding coupling lengths agree well with the experiments. This, in turn, verifies the validity of our theoretical model.

By varying the azimuthal angle *φ* in the sphere, we further obtain different spin–orbit Rabi oscillatory modes with relatively lower oscillating amplitudes in the same system (Fig. 5b). This is because the two vectors **B** and **S** are no longer perpendicular to each other. Particularly, if the state vector **S** is initially parallel to **B** [e.g., see the state vector at ($\theta =\pi /2,\varphi =0\,{\rm{or}}\,\pi$)], it does not undergo any precession, and eventually, Rabi oscillation disappears completely.

### Electrically controlled synthetic magnetic field

**Fig. 6:**
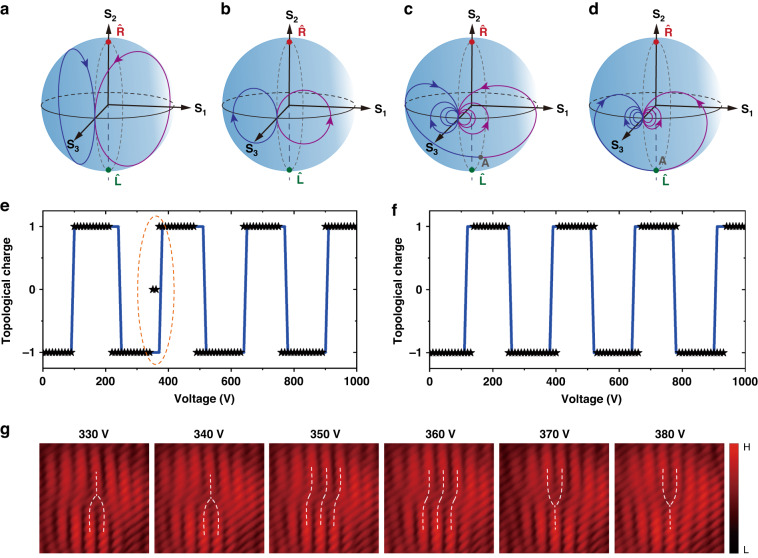
Tunable emissions of OAM beams. This is achieved by electrically tuning the phase mismatch quantity in a c-cut electro-optic lithium niobate crystal. **a**, **b** The voltage-dependent Rabi oscillatory modes achieved with an initial state $(\theta =\pi /2,\varphi =\pi /2)$ and a beam width *r*_0_ = 4 μm: **a,** *V* = ±50 V ($\triangle \beta =\mp 40.4{{\mathbf{m}}}^{{-}1}$); **b**, V = ±200 V ($\triangle \beta =\mp 161{{\rm{m}}}^{{-}1}$). **c**, The motion trajectory (blue line) of the spinor, which moves slowly from point A to its initial position $(\theta =\pi /2,\varphi =\pi /2)$ by gradually sweeping the voltage from 0 to 1000 V; while altering the voltage sign producing a symmetric trajectory (purple line). **d** The same as described in **c** but in the case of *r*_0_ = 5 μm. **e**, **f** The topological charge as a function of voltage after a coupling length *z* = 30 mm: **e** *r*_0_ = 4 μm; **f** *r*_0_ = 5 μm. The blue curves denote theoretical results, while the black data points indicate experiments. **g** The measured interferograms show a transition of the topological structure of light near *V* = 350 V, as highlighted by an ellipse in **e**

We introduce another tunable degree, allowing us to engineer the synthesized magnetic field by electrically modulating the phase mismatch in the crystal. This offers another knob to flexibly manipulate the spin–orbit Rabi oscillations. To demonstrate this possibility, we consider electrically tuning the phase mismatch quantity in a c-cut electro-optic lithium niobate (LN) crystal ($\bar{\gamma }=-0.08$). Considering a transverse modulation, the principal refractive index is given by^54^ ${n}_{x}={n}_{o}+0.5{n}_{o}^{3}{\gamma }_{22}V/d$, ${n}_{y}={n}_{o}-0.5{n}_{o}^{3}{\gamma }_{22}V/d$, and ${n}_{z}={n}_{e}$, respectively, where *n*_*o*_ and *n*_*e*_ denote the ordinary and extraordinary refractive indexes of the LN crystal, respectively, in the absence of an electrical field, *V* is the applied voltage, and *d* is the thickness of the crystal. Here *γ*_22_ = 6.8 pm/V is an electro-optic coefficient. In this platform, the knob *V* is utilized to finely tune the phase mismatch parameter $\triangle \beta =-{k}_{0}{n}_{o}^{3}{\gamma }_{22}V/d$ and the synthetic magnetic field **B** is changed accordingly. We, therefore, obtain a voltage-dependent transition between different Rabi oscillatory modes in the phase mismatching regime. Given a beam width *r*_0_ = 4 μm, we present two oscillatory modes at the voltages *V* = ±50 V, corresponding to phase mismatch quantities $\triangle \beta =$ ∓$40.4\,{{\rm{m}}}^{{-}1}$ (Fig. 6a). The system supports phase mismatching Rabi oscillations with less pronounced amplitudes than those in the phase matching condition. As the voltage is increased to *V* = ±200 V ($\triangle \beta =\mp 161\,{{\rm{m}}}^{{-}1}$), the strong detuned effect enhances the Rabi frequency but decreases the oscillation amplitude (Fig. 6b). This is due to the fact that the Rabi frequency only depends on the magnetic field strength; whereas the spin-orbit coupling relies on both the magnetization orientation and strength.

We employ this property and demonstrate an output of an orbital-angular-momentum beam with electrically tunable topological structures. We set the coupling length of the LN crystal to 30 mm. When there is no voltage applied to it, the effect of phase matching (resonant) driving field causes the original spinor to evolve to a certain point A on the higher-order Poincaré sphere (Fig. 6c). The state situated at point A generally exhibits a noncanonical vortex with non-zero orbital angular momentum. As the voltage is slowly increased from 0 to 1000 V, the system becomes phase mismatching, and the resulting quantity ∆*β* varies gradually from 0 to −807 m^−1^. As a result, the output state begins to move slowly with the voltage from point A toward its origin, following a spiral motion trajectory on the sphere (Fig. 6c). Altering the sign of the voltage leads to a symmetric trajectory about a longitude line at $\varphi =\pi /2$. Similar voltage-dependent spinor behavior is observed for another beam width *r*_0_ = 5 μm (Fig. 6d). Based on this, gradually varying *V* can effectively shift the spinor back and forth between the northern and southern hemispheres, resulting in a variation of the wavefront chirality with the topological charge being either 1 or −1. Note that our tunable system is not limited to the first-order sphere. A similar result for the higher-order topological structures is expected; see Supplementary Section D. Therefore, it is readily extended to the higher-order sphere, leading to OAM beam emissions with larger tunable topological charges. This external control knob allows us to observe the transition between different topological structures of light. Figure 6e, f presents the relationships between the topological charge and the voltage for the two different beam parameters.

We perform experiments to observe this dynamic process. We hence prepare the tunable system (Supplementary Section E) and gradually increase the voltage at an interval of ∆*V* = 10 V. The topological wavefront of light is experimentally identified by the interferogram between light emitting from the crystal and the reference plane wave. Figure 6e, f shows the measured voltage-dependent topological charges (black data points) both for *r*_0_ = 4 μm and *r*_0_ = 5 μm, respectively, which approximately match the theoretical results (blue lines). Both results reveal periodic transitions between two different topological structures with nearly the same transition period. The interpretation of the similar period is the flat (weak) spin-orbit coupling regime where the magnetic field is slowly varying with *r*_0_. Figure 6g shows a transition of topological structures of light from a negative charge (${\mathscr{l}}=-1$) to a positive charge (${\mathscr{l}}=+1$) by sweeping the voltage from 330 to 380 V. It is important to note that this significant topological transition is a result of a slight change of refractive index modulation $\triangle n=\left|{n}_{y}-{n}_{x}\right|=2.44\times {10}^{-6}$ induced by a voltage change of ∆*V* = 30 V. This ultrahigh sensitivity near the transition points might find relevant applications in precision measurements with the ‘two-level’ platform.

## Discussion

To summarize, we reported the first demonstration of spin–orbit-coupled Rabi oscillations in an effective two-level system driven by an engineered synthetic magnetic field. We constituted the pseudo spin-1/2 model in the higher-order regime, where the right- and left-handed circularly polarized vortex beams represent the spin-up and spin-down equivalents, respectively. Different degrees of freedom were exploited to fully engineer the synthetic magnetic field and hence to control the Rabi oscillations both in the phase matching and mismatching regimes. Particularly, we introduced the beam structure to effectively engineer the magnetization vectors, opening up efficient ways to control Rabi oscillations in the weak and strong coupling regimes. This is fundamentally different from those relying on material structures^30–37^. We further demonstrated electrically controlled synthetic magnetism, allowing us to finely control the Rabi transitions between different oscillatory modes. This enables an output of an orbital-angular-momentum beam with electrically tunable topological structures. We believe the presented formalism constitutes a general framework to explore the spin-orbit couplings using the synthesized magnetic field.

We note that manipulating structured light with both the spin and orbital angular momentum has drawn considerable interest^27,39,41^. Previous work mainly concentrates on two-dimensional transverse light fields. Manipulating the spin-orbit angular momentum beams in three or even higher dimensions is still challenging^39^. Our demonstration, however, allows full control of the spin–orbit state of light not only in the transverse plane but also along the propagation direction. It hence provides routes to simultaneously manipulating the spin and orbital angular momentum in (2 + 1)D configuration. If considering a spatiotemporally modulated crystal, the spacetime-dependent magnetization vector enables four-dimensional control of spin–orbit light beams, which we have discussed. Hence this work offers a new platform for three or even higher-dimensional manipulation of structured light.

Finally, our system is equivalent to the settings described by the Pauli equation, allowing us to study intriguing analogous phenomena accompanied by spinor evolution, such as the topological Hall effect and Stern–Gerlach effect, as already demonstrated with the Pauli equation in quantum mechanics^52,53^ and its equivalent in nonlinear optics^32–35^. These are possible in our higher-order platform driven by an appropriately designed magnetization vector, which can originate from a controllable light-crystal detuning or the laser beam’s structures. The presented framework connects the field of spintronics, nonlinear optics, and higher-order optics studied here, opening new possibilities for spinor manipulation in the higher-order regime, which can find applications in information processing.

## Materials and methods

### Initial spinor preparation

We consider using a standard q-plate to experimentally generate an initial spinor that is mapped onto the equator of the first-order (${\mathscr{l}}=1$) Poincaré sphere. In our experiments, a nematic liquid crystal is used to construct the q-plate with a topological charge of $q=1/2$. Specifically, the nematic liquid crystal is sandwiched between two planar glasses, forming a 2 × 2 mm planar cell. The liquid crystal thickness is chosen as 6 μm such that the half-wave retardation between two orthogonal polarization components is realized at the working wavelength of *λ* = 632.8 nm. A square signal with a voltage of 4.6 V and an AC frequency of 1 kHz is applied to modulate the liquid crystal. The constructed q-plate is used to convert a linearly polarized incident beam into the spin–orbit (vector–vortex) light beam. The conversion efficiency is about 97% at *λ* = 632.8 nm. Since the initial spinor contains equal weights on $\hat{R}$ and $\hat{L}$, both the spin and orbital angular momentum of the spinor are zeros, which facilitates the observation of the spin–orbit Rabi oscillations. The Jones matrix of the q-plate is written as

$$Q=\left[\begin{array}{cc}{{\cos }}(2\vartheta ) & {{\sin }}(2\vartheta )\\ {{\sin }}(2\vartheta ) & -{{\cos }}(2\vartheta )\end{array}\right]$$

where $\vartheta =q\phi +{\vartheta }_{0}$ denotes the orientation angle of the optical axis of the q-plate, with ${\vartheta }_{0}$ being a relatively initial angle. ${\vartheta }_{0}$ can be precisely controlled by rotating the q-plate with respect to the beam propagation direction. Here $\phi ={{\arctan }}(y/x)$. The q-plate is normally illuminated by a monochromatic laser beam whose polarization state can be expressed as $\eta =({\eta }_{x};{\eta }_{y})$. The output state of light from the q-plate takes the following form

$$\Phi =\left[\begin{array}{c}{{\cos }}\left(2\vartheta \right){\eta }_{x}+{{\sin }}\left(2\vartheta \right){\eta }_{y}\\ {{\sin }}\left(2\vartheta \right){\eta }_{x}-{{\cos }}\left(2\vartheta \right){\eta }_{y}\end{array}\right]$$

It is characterized by a topological number *q* and is featured by a spatial distribution with varying polarization. Since the topological number of the q-plate is chosen as $q=1/2$, a complex spinor mapping in the first-order Poincaré sphere is achieved. Particularly, considering the incident beam with horizontal polarization, i.e., *η* = (1; 0), Φ is reduced to

$$\Phi ={{\cos }}\left(\phi +\varphi \right)\hat{x}+{{\sin }}(\phi +\varphi )\hat{y}$$

where we have defined $\varphi =2{\vartheta }_{0}$. Clearly, this result is in accordance with Eq. (1) when letting $\theta =\pi /2$. Changing the orientation angle ${\vartheta }_{0}$, all possible spinors mapped on the equator of the first-order Poincaré sphere is achievable.

### Self-created element for generating the Bessel beams

In experiments, we utilize a self-created sharp-edge element for generating the nondiffracting Bessel-structured light beam. This requires that the diffractive waves coming from the sharp-edge element should be in phase, i.e., their high-spatial-frequency wavevectors exhibit circular symmetry in the reciprocal space. We realize this phase distribution of the diffractive wavevectors using an ultrathin metallic disc with a radius denoting as *ρ*. The disc is deposited onto a glass substrate. In element fabrication, we use a 50-nm-thick gold film initially deposited onto a 0.3-mm-thick substrate. A chromium film of 10 nm thickness is deposited as an adhesion layer. The UV lithography, together with the ion beam, is then used to form a disc on the metallic surface. Supplementary Section A provides a detailed fabrication procedure of the metallic disc. The incident beam is truncated sharply at the element edge, which induces significant in-phase diffractive waves, generating the nondiffracting Bessel beam.

We mention that there are many techniques, such as using conventional axicons or circular rings (together with a Fourier lens) to generate the nondiffracting Bessel beams. Regarding this issue, our technique has the following advantages. Firstly, the generated Bessel beam size is closely related to the radius *ρ*, enabling us to realize the Bessel beam with tunable width. This is more convenient for us to study the beam-dependent spin–orbit couplings. Secondly, the sharp-edge diffraction of the element induces significant high-spatial-frequency waves, allowing to generation of the spin–orbit Bessel beam at the deep-subwavelength scale. According to a definition of the numerical aperture for the nondiffracting first-order Bessel beam, i.e., ${\rm{NA}}=0.292\lambda /W$ (see ref. ^55^), where *W* represents the full width at half maximum of the main lobe, we calculate the resulting numerical aperture of the element as NA ≈0.8 (here *W* = 0.36*λ*). Such a high NA remains difficult to be achieved with conventional axicon devices and other techniques. Therefore, our self-created element provides a practicable method to generate the nondiffracting spin–orbit beams, enabling us to observe the spin-orbit Rabi oscillations both in the strong and weak coupling regimes.

### Experimental alignment of the crystal

The alignment of the crystal is essential in the experiment since the spin–orbit Rabi oscillation requires that the propagation direction of the spin–orbit light beam should coincide with the optical axis of the crystal. To address the alignment, firstly, the laser beam is precisely collimated by moving the CCD along the optical axis over a sufficient distance. We then use the collimated laser to align the crystal that is placed onto a stage. The position of the stage can be precisely adjusted in three dimensions. If the crystal is aligned, the extraordinary and ordinary beams overlap during propagation, resulting in a symmetric doughnut-shape distribution; If the crystal is not aligned, due to the birefringent effect, these two beams slightly separate in space, resulting in a distorted light pattern. These processes can be monitored using a high-resolution (pixel size is 1.4 μm) CCD. The alignment precision depends on the maximum separation ∆*r* and the crystal length *L*. Note that ∆*r* is relevant to the choice of beam width. For example, considering a Bessel–Gaussian beam width *r*_0_ = 3.5 μm, the maximum separation can be calculated as ∆*r* = 8 μm after a coupling length of *L* = 30 mm. As a result, the corresponding angle between the two beams is estimated as ∆*r*/*L* = 8 μm/30 mm. Whereas for a beam width of *r*_0_ = 15 μm, the angle increases to ∆*r*/*L* = 30 μm/30 mm.

### Derivation of the Pauli equation

We constitute an equivalent of the two-level system in the higher-order regime of light and introduce the analogous Pauli equation in the presence of a synthetic magnetic field. We achieve this by studying light-crystal interaction in the linear regime, with a spatially structured mutual beam comprising the superposition of two pure spin–orbit states $\hat{R}$ and $\hat{L}$, as defined in Eq. (1) of the manuscript. It is represented geometrically by the higher-order Poincaré sphere. Owing to the vectorial nature of the spin–orbit light beam, the crystal plays as a space-varying retarder in the realm of spin–orbit coupling^56^. We investigate this problem using the Maxwell theory. Specifically, we start by considering the following vector-wave equation

$$-\nabla \times \left[{\mu }^{-1}\cdot \left(\nabla \times \widetilde{{\bf{E}}}\right)\right]=\hat{\epsilon }\cdot \frac{{\partial }^{2}\widetilde{{\bf{E}}}}{\partial {t}^{2}}$$

where $\widetilde{{\bf{E}}}$ is the spatiotemporal light field, $\hat{\epsilon }$ and *μ* denote the dielectric tensor and the permeability of the crystal, respectively. *t* is elapsed time. We concentrate on the spatial effect of spin–orbit coupling and hence separate the space and time variables in the spatiotemporal Maxwell equation by expressing the complex light field in a form like $\hat{{\bf{E}}}\left(x,y,z,t\right)={\bf{E}}\left(x,y,z\right){{\exp }}(-i\omega t)$, where *ω* is the carrier-wave frequency and ${\bf{E}}={E}_{x}\hat{x}+{E}_{y}\hat{y}+{E}_{z}\hat{z}$ denotes the complex amplitude. In this case, a three-component coupled-wave equation represented in the Cartesian coordinate system is obtained, written as follows:

$$\begin{array}{c}{\nabla }^{2}{E}_{x}+{\beta }_{x}^{2}{E}_{x}=\frac{\partial }{\partial x}(\nabla \cdot {\bf{E}})\\ {\nabla }^{2}{E}_{y}+{\beta }_{y}^{2}{E}_{y}=\frac{\partial }{\partial y}(\nabla \cdot {\bf{E}})\\ {\nabla }^{2}{E}_{z}+{\beta }_{z}^{2}{E}_{z}=\frac{\partial }{\partial z}(\nabla \cdot {\bf{E}})\end{array}$$

where ${\beta }_{j}=\omega \sqrt{\mu {\epsilon }_{j}}$ (*j* = *x*, *y*, *z*) is a propagation constant of wave component *E*_*j*_ in the crystal. Note that we have assumed that light propagates along the optical axis of the crystal, which is denoted as the *z*-axis. Owing to the non-zero term $\nabla \cdot {\bf{E}}\,\ne \,0$ in the crystal, the spin–orbit coupling takes place and has a nontrivial influence on beam propagation. We further write the solution of the coupled-wave equation as follows:

$$\begin{array}{c}{E}_{x}\left(x,y,z\right)={A}_{x}\left(x,y,z\right){{\exp }}(i{\beta }_{x}z)\\ {E}_{y}\left(x,y,z\right)={A}_{y}\left(x,y,z\right){{\exp }}(i{\beta }_{y}z)\\ {E}_{z}\left(x,y,z\right)={A}_{z}\left(x,y,z\right){{\exp }}(i{\beta }_{z}z)\end{array}$$

where ${\bf{A}}={A}_{x}\hat{x}+{A}_{y}\hat{y}+{A}_{z}\hat{z}$ is a spatially distributed complex envelope varying slowly along with *z*, i.e., ${\partial }^{2}{A}_{x}/\partial {z}^{2}\ll {\beta }_{x}\frac{\partial {A}_{x}}{\partial z}$ and ${\partial }^{2}{A}_{y}/\partial {z}^{2}\ll {\beta }_{y}\frac{\partial {A}_{y}}{\partial z}$. With these conditions, we reduce the coupled-wave equation to

$$\begin{array}{c}\left({\nabla }_{\perp }^{2}{A}_{x}+i2{\beta }_{x}\frac{\partial {A}_{x}}{\partial z}\right){{\exp }}\left(i{\beta }_{x}z\right)=\frac{\partial }{\partial x}(\nabla \cdot {\bf{E}})\\ \left({\nabla }_{\perp }^{2}{A}_{y}+i2{\beta }_{y}\frac{\partial {A}_{y}}{\partial z}\right){{\exp }}\left(i{\beta }_{y}z\right)=\frac{\partial }{\partial y}(\nabla \cdot {\bf{E}})\end{array}$$

where ${\nabla }_{\perp }^{2}={\nabla }_{{xx}}+{\nabla }_{{yy}}$ is a transverse momentum operator. It is clear that the crystal’s anisotropy is the origin of the spin–orbit coupling. We expand the term $\nabla \cdot {\bf{E}}$, and consider the following constraint condition:

$${\epsilon }_{x}\frac{\partial {E}_{x}}{\partial x}+{\epsilon }_{y}\frac{\partial {E}_{y}}{\partial y}+{\epsilon }_{z}\frac{\partial {E}_{z}}{\partial z}=0$$

In this manner, a general Schrödinger-like equation governing the propagation dynamics of light is obtained as

$$i2\bar{\beta }\frac{\partial }{\partial z}\left(\begin{array}{c}{A}_{x}\\ {A}_{y}\end{array}\right)=\left[\begin{array}{cc}-{\nabla }_{\perp }^{2}+\bar{\gamma }{\nabla }_{{xx}} & \bar{\gamma }{\nabla }_{{yx}}{{\exp }}(+i\triangle \beta \cdot z)\\ \bar{\gamma }{\nabla }_{{xy}}{{\exp }}(-i\triangle \beta \cdot z) & -{\nabla }_{\perp }^{2}+\bar{\gamma }{\nabla }_{{yy}}\end{array}\right]\left(\begin{array}{c}{A}_{x}\\ {A}_{y}\end{array}\right)$$

where we have assumed that $\bar{\beta }=({\beta }_{x}+{\beta }_{y})/2$ and $\bar{\gamma }=({\gamma }_{x}+{\gamma }_{y})/2$, for the shallow linear birefringence, with ${\gamma }_{j}=1-{\epsilon }_{j}/{\epsilon }_{z}$ describing the anisotropic degree of the crystal. $\triangle \beta ={\beta }_{y}-{\beta }_{x}$ is a phase mismatch quantity arising from the beating between the two components. The Hamiltonian is related to spatial gradient operators: ${\nabla }_{{xy}}={\partial }^{2}/(\partial x\partial y)$ and ${\nabla }_{{yx}}={\partial }^{2}/(\partial y\partial x)$ (they have property ${\nabla }_{{xy}}={\nabla }_{{yx}}$). Note that the transverse momentum operator ${\nabla }_{\perp }^{2}$ plays a role in the diffraction effect of the wave, which is analogous to the temporal decoherence effect appearing in the quantum system. We further transform *A*_*x*_ and *A*_*y*_ to the rotating frame by defining

$$\begin{array}{c}{A}_{x}\left(x,y\right)={{\exp }}(+i\triangle \beta \cdot z/2){\widetilde{A}}_{x}(x,y)\\ {A}_{y}\left(x,y\right)={{\exp }}(-i\triangle \beta \cdot z/2){\widetilde{A}}_{y}(x,y)\end{array}$$

respectively. In the rotating frame, the Schrödinger-like equation is modified as

$$i2\bar{\beta }\frac{\partial }{\partial z}\left(\begin{array}{c}{\widetilde{A}}_{x}\\ {\widetilde{A}}_{y}\end{array}\right)=\left[\begin{array}{cc}\left(-{\nabla }_{\perp }^{2}+\bar{\gamma }{\nabla }_{{xx}}\right)+\bar{\beta }\triangle \beta & \bar{\gamma }{\nabla }_{{yx}}\\ \bar{\gamma }{\nabla }_{{xy}} & \left(-{\nabla }_{\perp }^{2}+\bar{\gamma }{\nabla }_{{yy}}\right)-\bar{\beta }\triangle \beta \end{array}\right]\left(\begin{array}{c}{\widetilde{A}}_{x}\\ {\widetilde{A}}_{y}\end{array}\right)$$

To obtain an equivalent of the Pauli equation, which describes the spinnings of a quantum particle in a driven magnetic field, it is relevant to transform the present setting from the Cartesian basis to a circular basis by employing a pseudo spin description. We therefore identify $\hat{R}$ and $\hat{L}$ as the pseudo-spin-up and pseudo-spin-down components of a spin-orbit-coupled spinor, respectively. The complex spinor carried by the envelope $\widetilde{{\bf{A}}}(x,y,z)$

$$\widetilde{{\bf{A}}}\left(x,y,z\right)={\widetilde{A}}_{0}\left(x,y,z\right)[{\Phi }_{R}\left(z\right)\hat{R}+{\Phi }_{L}(z)\hat{L}]$$

comprises a superposition of $\hat{R}$ and $\hat{L}$ with different weights, which can be expressed in a normalized form as ${\Phi }_{R}\left(z\right)={{\sin }}\left[\theta \left(z\right)/2\right]{{\exp }}[+i\varphi \left(z\right)/2]$ and ${\Phi }_{L}\left(z\right)={{\cos }}\left[\theta \left(z\right)/2\right]{{\exp }}[-i\varphi \left(z\right)/2]$, respectively. Substituting the expression of $\widetilde{{\bf{A}}}\left(x,y,z\right)$ into the Schrödinger-like equation and using a transformation matrix ${\rm{T}}=[1,-{i;}\,1,i]$, we obtain the equivalent of the Pauli equation written as a spin-1/2 form

$$i\frac{\partial }{\partial z}\left[\begin{array}{c}{\Phi }_{R}\left(z\right)\\ {\Phi }_{L}\left(z\right)\end{array}\right]=\left(\frac{1}{2{\bf{M}}}{\nabla }_{\perp }^{2}{\widetilde{A}}_{0}-\frac{1}{2}\sigma \cdot {\bf{B}}\right)\left[\begin{array}{c}{\Phi }_{R}\left(z\right)\\ {\Phi }_{L}\left(z\right)\end{array}\right]$$

where ${\bf{M}}=2\bar{\beta }{\widetilde{A}}_{0}/(\bar{\gamma }-2)\cdot {\bf{I}}$ is the equivalent mass of the spinor with **I** being the 2 × 2 identity operator and *σ* is the Pauli matrix vector $\sigma =({\sigma }_{1},{\sigma }_{2},{\sigma }_{3})$, defined in the circular basis as

$${\sigma }_{1}=\left[\begin{array}{cc}0 & -i\\ i & 0\end{array}\right],{\sigma }_{2}=\left[\begin{array}{cc}1 & 0\\ 0 & -1\end{array}\right],{\sigma }_{3}=\left[\begin{array}{cc}0 & 1\\ 1 & 0\end{array}\right]$$

Here **B** is a magnetic field equivalent presented in the rotating frame. It can be expressed as ${\bf{B}}=({B}_{1},{B}_{2},{B}_{3})$, where

$${B}_{1}=-\Omega ,{B}_{2}=0,{B}_{3}=-\triangle {\rm{\beta }}$$

and

$$\Omega =\frac{\bar{\gamma }}{\bar{\beta }}\frac{\iint \left|{\nabla }_{{xy}}{\widetilde{A}}_{0}\left(x,y\right)\right|{dxdy}}{\iint \left|{\widetilde{A}}_{0}\left(x,y\right)\right|{dxdy}}$$

Clearly, the synthetic magnetic field is closely related to a spatial gradient of the light field and the inherent phase mismatch quantity. It indicates that the synthetic magnetic field can be controlled either by engineering the phase mismatch ∆*β* in the crystal or by structuring the light field ${\widetilde{A}}_{0}\left(x,y\right)$ in space. This engineered magnetization vector **B** leads to observations and controls of a new form of spin–orbit Rabi oscillations, as demonstrated in this work.

### Supplementary information

Supplementary
