# Supplementary material for: Spin-orbit Rabi oscillations in optically synthesized magnetic fields
Source: Light Sci Appl. 2023 Aug 28;12:205. doi: 10.1038/s41377-023-01238-8 (PMC10462765; doi:10.1038/s41377-023-01238-8)
Supplement: Supplementary file 1 — Supplementary [file 41377_2023_1238_MOESM1_ESM.pdf]

Supplementary Information for
"Spin-orbit Rabi oscillations in optically synthesized magnetic fields"

Guohua Liu[1,2], Xiliang Zhang[1,2], Xin Zhang[1,2], Yanwen Hu[1,2], Zhen Li[1,2,3*], Zhenqiang Chen[1,2,3], and Shenhe Fu[1,2,3*]

[1]*Department of Optoelectronic Engineering, Jinan University, Guangzhou 510632, China*

[2]*Guangdong Provincial Key Laboratory of Optical Fiber Sensing and Communications, Guangzhou 510632, China*

[3]*Guangdong Provincial Engineering Research Center of Crystal and Laser Technology, Guangzhou 510632, China*

[*]*Corresponding authors: fushenhe@jnu.edu.cn; ailz268@126.com*

Section A: Fabrication procedure of the self-created sharp-edge element

In element fabrication, a 50-nm-thick gold film is considered to be initially deposited onto a 0.3-mm-thick substrate. A chromium film of 10 nm thickness is deposited as an adhesion layer. Figure S1**a** and S1**b** (top view of the sample) show the schematic diagram of the metallic disc. In experiments, the sharp-edge discs with two different cases of radius (ρ = 400 μm and ρ = 7.5 μm) are fabricated for generating the nondiffracting Bessel structured beams in the micro- and nano-scale, as shown by their optical microscopic images in Fig. S1**c** and S1**d**, respectively. In the following, we introduce a complete procedure for the sample fabrication, see Fig. S1**e**. Firstly, the 10-nm-thick chromium film (adhesion layer) and the 50-nm-thick Au film are successively deposited onto a clean 0.3-mm-thick glass substrate using the physical vapor deposition (PVD) technique. After PVD, the 1.2-μm-thick positive photoresist (AZ5214) is spin-coated onto the metallic film and baked at 100 ℃ for 2 minutes. Note that baking can evaporate the solvent in the photoresist and increase the viscosity of the photoresist. Thirdly, a prepared mask is aligned carefully and placed on the photoresist. A UV light source is utilized to illuminate the sample for 30 seconds and develop for 1 minute. The pattern (disc) of the mask is transferred to the photoresist after UV exposure. Finally, the ion beam is used to etch and peel off the metallic films outside the pattern. The etched sample is placed in an acetone solution to dissolve the photoresist and then obtain the final desired metallic disc.

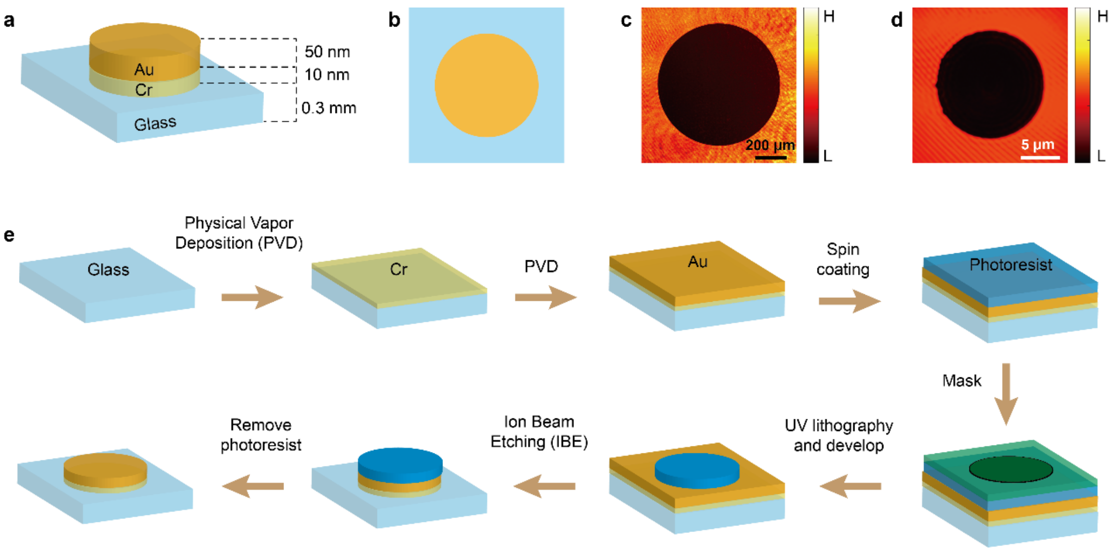

FIG. S1: **Fabrication of the self-created sharp-edge element**. **a**, The schematic diagram of metallic disc, which consists of 50-nm-thick gold film, 10-nm-thick chromium film (as an adhesion layer) and 0.3-mm-thick substrate. **b**, Top view of disc. **c**, **d**, Images of two discs with different radius: **c**, ρ = 400 μm and **d**, ρ = 7.5 μm. **e**, Complete fabrication procedures.

Section B: Observation of the spin angular momentum oscillations

We base on the experimental setup presented in Fig. 2**a** in the main text, demonstrating the SAM oscillation in the weak coupling regime. This is manifested by the z-dependent variations of the circular polarization components of light. By rotating the QWP to +45° and -45° with respect to the x axis, respectively, the left-handed and right-handed circular polarization components can be extracted from the mutual beam at different coupling lengths. The experiment is performed using the same parameters as those in Fig. 3 of the main text. At the beginning, the state stays at the equator of the higher-order Poincaré sphere with equal weight on $\hat{R}$ and $\hat{L}$. The resulting right-handed and left-handed polarization components share identical intensity distributions (the first column of Fig. S2). With coupling lengths of z = 5 and 10 mm, the state evolves from the equator to the northern hemisphere (spin up). As a result, the left-handed component is partially converted to the right-handed counterpart. The intensity patterns in the second and third columns confirm these assertions, which show stronger intensity distributions in $\hat{R}$ than those in $\hat{L}$. Increasing the coupling length to z = 15 and 20 mm, the simulated and measured results suggest a reverse process (spin down) since the intensities of the left-handed components become stronger than those of the right-handed components. The measurements at z = 25 and 30 mm suggest a repeated process after a cycle evolution. The measured patterns indicate that the spinor becomes spin up again at these coupling lengths. These results, together with those shown in Fig. 3 of the main text, confirm that the given spinor indeed oscillates periodically along with the coupling length, featuring a spin-orbit Rabi oscillation.

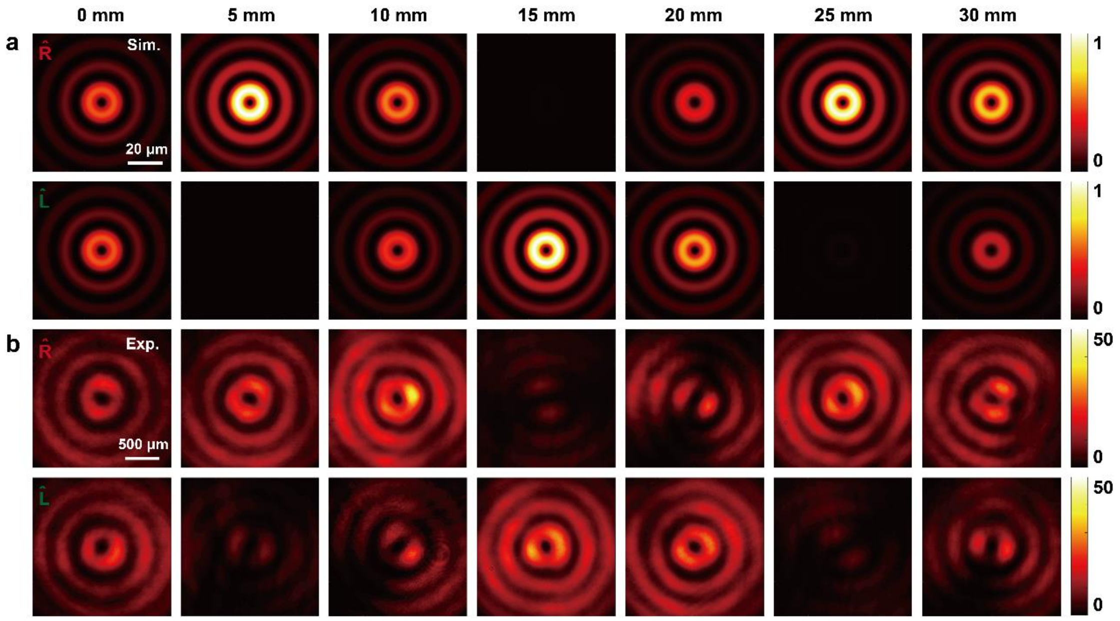

FIG. S2: **Observation of the SAM oscillation in the weak coupling regime**. **a**, **b**, The intensity distributions of the right- and left-handed circular polarization components at different coupling lengths: **a**, simulations; **b**, experiments. Panels in **a** (**b**) share the same scale, with the scale bar of 20 μm (500 μm). The experiments are carried out using the same parameters as those in Fig. 3 of the main text. The simulations are performed based on the Pauli equation (3) in the main text.

Section C: Spin-orbit Rabi oscillations with and without damping

A general harmonic oscillator for the spin-orbit Rabi oscillations actually can be identified from the Hamiltonian matrix of the system. As seen from the general dynamic equation [Eq. (2)] in the main text, the diagonal elements of the Hamiltonian matrix play an analogous decoherent effect of waves in the quantum system. In our case, it is the optical diffraction in space that results in a damping Rabi oscillation. However, if we consider a light beam without diffraction, the system can emulate a process for perfect Rabi oscillation. We perform simulations based on the general Eq. (2), by considering the initial condition: $\hat{\mathbf{A}}(r,\ z=0) = J_\ell(r/r_0)\Phi$, where J_ℓ is an ideal Bessel function of order ℓ and r_0 characterizes the beam width (here we set r_0 = 3.5 μm, in the weak spin-orbit coupling regime). The ideal Bessel beam that carries infinite energy is nondiffracting during interaction with the crystal, enabling a perfect Rabi oscillation without amplitude damping (see Fig. S3**a**). However, in practical experiment, we should slightly truncate the Bessel beam using an exponential or Gaussian window. The weakly truncated Bessel beam leads to an imperfect Rabi oscillation (see Fig. S3**b**). The weakly damping oscillatory amplitude is resulted from a slightly diffracting property of the truncated Bessel beam. It is important to note that a completely diffracting Laguerre-Gaussian beam having the same parameter r_0 cannot support Rabi oscillation, as shown in Fig. S3**c**.

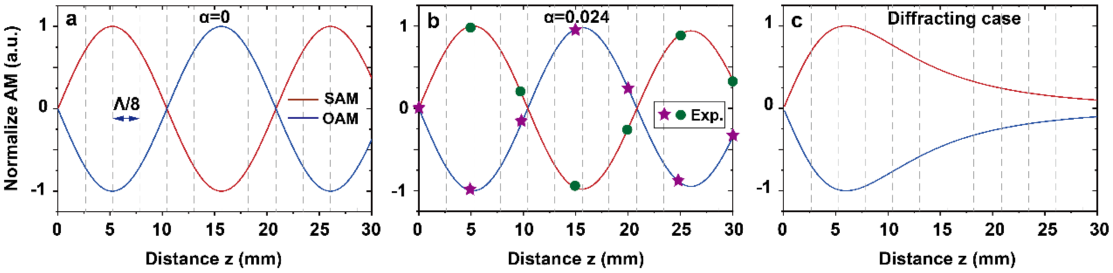

FIG. S3: **Perfect and imperfect Rabi oscillations**. **a**, Perfect spin-orbit Rabi oscillation achieved by a non-truncated Bessel beam (α = 0), where α is a beam truncation factor. This simulated result is in accordance with the theory shown in Fig. 5**c** of the main text. **b**, Weakly damping spin-orbit Rabi oscillation is achieved by a truncated Bessel beam (α = 0.024), in which case we have performed experiment with data indicated by purple stars and green points; **c**, By contrast, a diffracting Laguerre-Gaussian beam having initially beam width as the same with the Bessel beam width cannot support the spin-orbit Rabi oscillation. These panels display dynamical variations of the spin and orbital angular momentum along with the coupling length z. In simulations, the parameters are set the same as those in Fig. 3 of the main text.

Section D: Spinor oscillations with higher topological charges

For a fix crystal, the synthetic magnetic field, which determines the Rabi oscillation wavelength (frequency), is closely related to spatial gradient of the Bessel beam. Therefore, it is expected that varying the order of Bessel function, e.g., considering the beam with a higher topological charge, would lead to a change of the Rabi frequency. To see this effect, we numerically calculate the strength of the synthetic magnetic field with different topological charges, with result shown in Fig. S4**a**. Evidently, the increase of the topological charge slightly decreases the Rabi frequency, since a larger charge of spin-orbit beam leads to a relatively smaller spatial gradient. The simulated spinor oscillations with the coupling length (Fig. S4**b**) further confirm this effect.

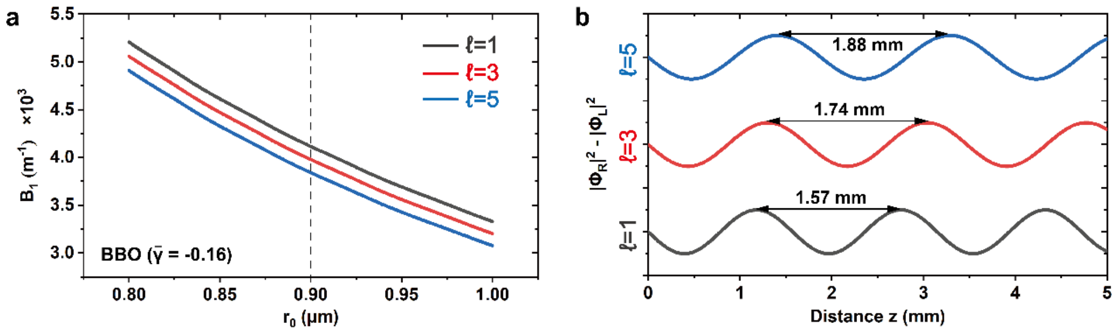

FIG. S4: **Spinor oscillations with higher topological charges**. **a**, The strength of synthetic magnetic field as a function of beam parameter r_0, for three different topological charges (ℓ = 1, 3, 5). The magnetic field is synthesized in the c-cut BBO crystal ($\bar{\gamma}$ = -0.16). **b**, Spinor oscillations with coupling length, for a fixed beam width r_0 = 0.9 μm (see dashed line in **a**).

Section E: An electrically controlled platform for tuning the synthetic magnetic field

The tunable platform is based on a c-cut lithium niobate (LN) crystal, in which we can electrically modulate the light-crystal detuning. This results in a tunable synthetic magnetic field. Figure S5 depicts the schematic light-crystal interaction process, controlled by an external voltage V. The transverse electrical modulation leads to a linear relation between the phase mismatch quantity and the applied voltage, that is $\Delta\beta = -k_0 n_o^3 \gamma_{22} V/d$, where k_0 is free-space wave number, n_o is refractive index of the ordinary beam, γ_{22} is an electro-optic coefficient, and d is the thickness of the LN crystal. This external knob allows us to flexibly engineer the synthetic magnetic field and hence to control the spin-orbit Rabi oscillations. Using this platform, we obtain a voltage-dependent transition between different Rabi oscillatory modes in the phase mismatching regime, resulting in an emission of OAM beams with topological charge being either 1 or -1. Note that our tunable system is not limited to the first-order sphere. It is readily extended to the higher-order sphere, leading to emissions of OAM beams with larger tunable topological charges (see section D).

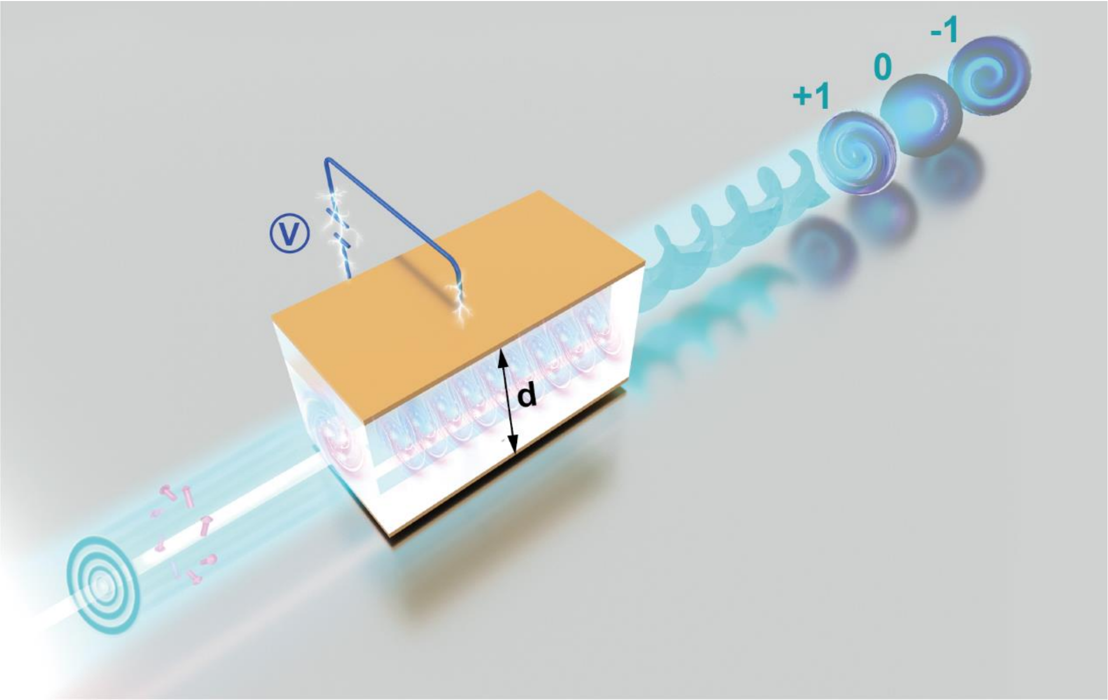

FIG. S5: **Schematic light-crystal interaction**. A spinor carried by the Bessel envelope is normally injected into the c-cut lithium niobate crystal, which is transversely modulated by an applied voltage. The spinor evolution trajectory in the crystal is controlled by the applied voltage, leading to transitions between different Rabi oscillatory modes. This enables an emission of OAM beams from the LN crystal with tunable topological charges.
